# Assessment of Quality and Readability of Information Provided by ChatGPT in Relation to Anterior Cruciate Ligament Injury

**DOI:** 10.3390/jpm14010104

**Published:** 2024-01-18

**Authors:** Stephen Fahy, Stephan Oehme, Danko Milinkovic, Tobias Jung, Benjamin Bartek

**Affiliations:** Centrum für Muskuloskeletale Chirurgie, Charité Universitätsmedizin, 10117 Berlin, Germany; stephan.oehme@charite.de (S.O.); tobias.jung@charite.de (T.J.); benjamin.bartek@charite.de (B.B.)

**Keywords:** anterior cruciate ligament (ACL), ACL reconstruction surgery (ACL-R), health literacy, readability, ChatGPT, artificial intelligence (AI), orthopaedic injuries, DISCERN criteria, natural language processing, patient education materials (PEMS)

## Abstract

The aim of our study was to evaluate the potential role of Artificial Intelligence tools like ChatGPT in patient education. To do this, we assessed both the quality and readability of information provided by ChatGPT 3.5 and 4 in relation to Anterior Cruciate Ligament (ACL) injury and treatment. ChatGPT 3.5 and 4 were used to answer common patient queries relating to ACL injuries and treatment. The quality of the information was assessed using the DISCERN criteria. Readability was assessed with the use of seven readability formulae: the Flesch–Kincaid Reading Grade Level, the Flesch Reading Ease Score, the Raygor Estimate, the SMOG, the Fry, the FORCAST, and the Gunning Fog. The mean reading grade level (RGL) was compared with the recommended 8th-grade reading level, the mean RGL among adults in America. The perceived quality and mean RGL of answers given by both ChatGPT 3.5 and 4 was also compared. Both ChatGPT 3.5 and 4 yielded DISCERN scores suggesting “good” quality of information, with ChatGPT 4 slightly outperforming 3.5. However, readability levels for both versions significantly exceeded the average 8th-grade reading level for American patients. ChatGPT 3.5 had a mean RGL of 18.08, while the mean RGL of ChatGPT 4 was 17.9, exceeding the average American reading grade level by 10.08 grade levels and 9.09 grade levels, respectively. While ChatGPT can provide both reliable and good quality information on ACL injuries and treatment options, the readability of the content may limit its utility. Additionally, the consistent lack of source citation represents a significant area of concern for patients and clinicians alike. If AI is to play a role in patient education, it must reliably produce information which is accurate, easily comprehensible, and clearly sourced.

## 1. Introduction

With an estimated incidence of 200,000 Anterior Cruciate Ligament (ACL) ruptures per annum in the USA and over 40,000 ACL ruptures reported yearly in Germany, ACL injuries represent a significant burden on healthcare systems across the world [1,2,3]. Globally, ACL reconstruction surgery has seen a marked increase in frequency in the last decade [4]. However, Anterior Cruciate Ligament Reconstruction (ACL-R) is not the correct decision for every patient, with the optimal treatment being contingent on multiple patient factors, including age, comorbidities, functional demands, occupation, desired activity level, and patient preference. Young and physically active patients undergoing ACL-R report lower subjective instability and a higher return to play rates than conservatively managed patients, However, in cohorts of patients with low functional demands, low physical activity levels, and low motivation levels, conservative therapy yields broadly comparable results as operative intervention [5]. Additionally, the choice of graft used in ACL-R influences surgical technique, potential complications, and functional outcomes. Optimal graft selection is not only dependent on graft properties but is also influenced by patient characteristics and expectations. As such, the appropriate therapy choice is both multifactorial and highly individualised.

The highly individualized nature of treatment following ACL injury means that patient education and shared-decision making is of upmost importance. In modern times, the widespread availability of both the Internet and smartphones has made access to medical education resources far easier, and this in turn has facilitated a shift from the traditional, clinician-led, paternalistic decision-making model to a shared-decision making model between patient and clinician. Patients have a strong desire to be informed, heard, and involved in the decision-making process regarding their treatment options [6]. In order to play a part in the decision-making process patients must be able to “obtain and interpret medical information and to, in turn, use this information with sufficient competence to enhance health”; this is referred to as health literacy [7]. A key tenet of health literacy is that patients can read and understand the resources available to them. With the average American reading at an 8th grade level (13–14 years old) expert groups recommend that patient education resources should not be written above a 6th grade level (11 to 12 years old) to optimise readability [8,9,10,11]. Despite this, numerous studies have found that patient education materials (PEMS) are often written at reading grade levels (RGLs) far above those recommended, making them of limited utility for patients in their decision-making processes [12,13,14,15].

The majority of orthopaedic patients cite the internet as a valuable resource for patient education [13]. However, the most patients report using unreliable websites like Wikipedia to inform their treatment decisions [12]. Owing to the heavy reliance patients have on internet-based resources, clinicians should be able to refer patients to trusted websites which provide accurate information delivered at an appropriate RGL to optimize patient education. This in turn fosters improved health literacy and ultimately facilitates effective shared decision-making. The increasing popularity of Artificial Intelligence tools such as ChatGPT, both among the general population and also in the medical field could have positive implications for health literacy. Natural language processing tools, such as ChatGPT, have the potential to provide patients with easy and immediate access to highly individualised information, which may help to bridge the current knowledge gap between patient and clinician. However, to date, few studies have assessed the quality and readability of information provided by Artificial Intelligence (AI) tools like ChatGPT in relation to orthopaedic injuries [16]. As such, the aim of our study was to analyse both the quality and readability of information provided by ChatGPT relating to ACL injury and reconstruction. Additionally, we aimed to assess whether a significant difference existed between the quality and readability of answers given by both ChatGPT 3.5 and 4. ChatGPT 4 is the most up-to-date version of the software, offering a larger language model with more detailed response capabilities, and is deemed to represent a significant maturation in AI language modelling with the accuracy and reported problem solving capabilities on average 60% better than those ChatGPT 3.5 [17].

## 2. Materials and Methods

On 4 September 2023, the popular natural language processing tool, ChatGPT (OpenAI Global LLC, San Francisco, CA, USA) was used to answer common patient queries regarding Anterior Cruciate Ligament (ACL) injuries and prospective treatment options. These questions were derived from prior research that examined patient expectations in relation to ACL reconstruction surgery, as well as anecdotal evidence from frequently asked questions in our institution (see Appendix A) [18,19,20]. The questions were written at, or below, the average American reading level of 8th grade. The same questions were posed to both ChatGPT 3.5 and ChatGPT 4 and the responses were saved in Microsoft Word Documents. The quality of information provided by both ChatGPT 3.5 and 4 was assessed by the three named authors (SF, SO, DM), all of whom are registrars in orthopaedics, specializing in knee surgery.

The DISCERN criteria were used as the primary tool for the assessment of the quality of information. The DISCERN criteria comprise 16 questions, each rated on a 5-point scale, and are used to assess the quality of written health information [20]. The first eight questions refer to the reliability of the content produced by the authors and the next seven questions scrutinise treatment choices with the final question allowing for an overall rating. With a maximum score of 80, scores of 70 and above are deemed “excellent”, with scores of 50 and above being deemed as “good” [16].

To assess the readability of the answers given, the Readability Studio Professional Edition Program (Version 2021, Oleander Software Ltd., Vandalia, OH, USA) was used [21]. This software assesses readability using seven well-established assessment tools (Appendix B); the Flesch–Kincaid Reading Grade Level (FKGL), the Flesch Reading Ease Index (FRES), the SMOG score, the Fry Score, the Raygor Estimate the FORCAST score, and Gunning Fog score. Reading grade levels (RGLs) were expressed as a United States (US) grade level. The RGL is an estimate of the approximate level of education required to read and understand the content of a particular article [9,22,23]. The FRES Index expresses readability as an index score ranging from 0–100. Higher scores indicate an easier readability.

The inter-rater reliability (IRR) was calculated for the DISCERN criteria scores with a level of 0.81–1.00 being deemed as almost perfect agreement. The mean RGL for both ChatGPT 3.5 and 4 was compared with the recommended 8th grade reading levels using a one-sample Wilcoxon signed-rank test. Wilcoxon signed-rank test was also used to assess for statistically significant between-group differences in the mean total DISCERN criteria score and the mean score per DISCERN criteria category. All statistical analysis was performed using SPSS statistics for Windows, version 27 (IBM Corp, Armonk, NY, USA). This study did not require ethical approval as no patient-level data were used.

## 3. Results

The same 33 questions were posed to both ChatGPT 3.5 and 4 (see Appendix A). The questions were broadly grouped under the following headings: Treatment Choice, Short-Term Expectations, Long-Term Expectations, and Other. A typical example of answers provided by both ChatGPT 3.4 and ChatGPT 4 is provided below:

Question: Should I get ACL surgery?

*ChatGPT 3.5*

“I’m not a doctor, but I can provide some general information that might help you make a decision. Whether or not to get ACL reconstruction surgery depends on various factors such as the severity of your injury, your lifestyle, and your overall health. Here are a few things to consider:

- Consult a specialist: It’s important to consult with an orthopedic surgeon or sports medicine specialist who can thoroughly evaluate your condition. They will assess the extent of your injury and provide professional advice based on your individual circumstances.
- Lifestyle and activity level: If you lead an active lifestyle and participate in sports or activities that require a stable knee, ACL reconstruction surgery may be recommended. This surgery aims to restore stability to the knee and can help prevent further damage.
- Non-surgical options: In some cases, non-surgical treatments like physical therapy and rehabilitation exercises may be effective in improving the strength and stability of the knee. This approach is typically recommended for less severe injuries or for individuals with low activity levels.
- Risks and recovery: Like any surgical procedure, ACL reconstruction surgery has risks, including infection, blood clots, and adverse reactions to anesthesia. Additionally, the recovery period can be lengthy and require rehabilitation to regain strength and flexibility in the knee.

Ultimately, the decision to undergo ACL reconstruction surgery should be made in consultation with a medical professional who can evaluate your specific case. They will consider your goals, lifestyle, and overall health to guide you in making the best choice for your situation.”

*ChatGPT 4*

“I’m not a doctor, but I can provide some general information to help you make an informed decision. Whether or not you should get ACL reconstruction surgery depends on various factors, such as your age, overall health, activity level, and the severity of your ACL injury.

Here are a few things to consider when deciding whether to have ACL reconstruction surgery:

- Stability of the knee: If your ACL injury has caused instability in your knee, making it difficult to perform everyday activities or participate in sports, surgery may be recommended to restore stability.
- Activity level: If you lead an active lifestyle or participate in high-demand sports that require sudden pivoting, jumping, or cutting motions, surgery may be necessary to increase your chances of returning to your desired level of activity.
- Future complications: An untreated or inadequately treated ACL injury may lead to the development of secondary knee problems, such as meniscus tears or early-onset arthritis. Addressing the ACL injury with surgery can help reduce the risk of such complications.
- Non-surgical options: In some cases, non-surgical treatment, such as physical therapy and rehabilitation exercises, may be sufficient to regain knee strength and stability. This can be a viable option for individuals who have a less active lifestyle or engage in low-impact activities.
- Recovery and rehabilitation: ACL reconstruction surgery is followed by a period of recovery and rehabilitation, which can take several months. It’s important to consider the commitment, time, and effort required for rehabilitation before deciding on surgery.

To make an informed decision, it’s best to consult with a qualified orthopedic surgeon. They can evaluate your specific condition, discuss your goals and expectations, and recommend the most suitable treatment option for you.”

### 3.1. DISCERN Score

The mean DISCERN score for answers given by ChatGPT 3.5 was 55.4 (range, 52.2–58.5), from a maximum score of 80, with an ICC of 0.85 (Table 1). The mean DISCERN score for answers given by ChatGPT 4 was 62.09 (range 59.00–66.85) of a maximum score of 80, with an ICC of 0.91. No significant difference was observed between the mean DISCERN scores of ChatGPT 3 and 4. The mean score per DISCERN category (sixteen categories, maximum score of 5 per category) found a significant between-groups difference in favour of ChatGPT 4. ChatGPT 4 had a mean score of 3.86 (95% C.I. 3.79–3.93) compared with an average score of 3.46 with ChatGPT 3 (95% C.I. [3.40–3.54], *p* < 0.01) (Table 1). Regardless of the platform used, both ChatGPT 3.5 and 4 scored highly in categories relating to response relevance, and for consistently highlighting the importance of shared decision-making. Owing to a consistent lack of source citation both platforms consistently scored poorly in Questions 4 and 5, which relate to appropriate source citation.

### 3.2. Reading Grade Level

**Figure 1:**
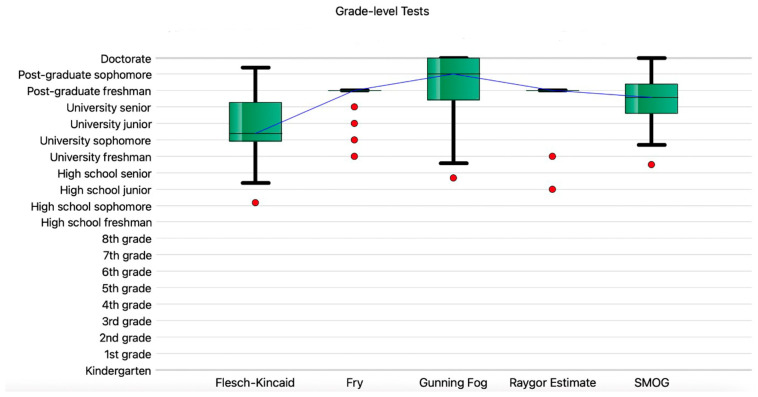
Reading grade level for ChatGPT 3.5. The horizontal line denotes the median; the upper and lower bounds of each box depict the interquartile range; whiskers show the lower and upper quartiles; circles indicate outliers.

**Figure 2:**
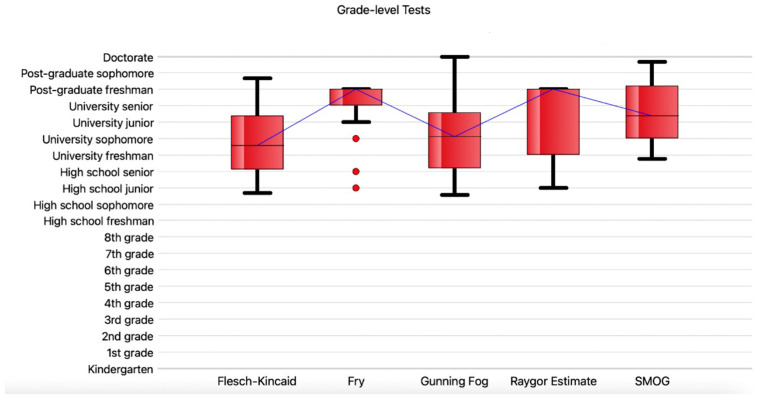
Reading grade level for ChatGPT 4. The horizontal line denotes the median; the upper and lower bounds of each box depict the interquartile range; whiskers show the lower and upper quartiles; circles indicate outlier.

The mean RGL of the questions posed by the investigators was 7.9 (range, 6–10.3), in keeping with the average 8th grade reading level of the general public in America. The mean RGL for answers given by ChatGPT 3.5 was 18.08 (range, 14.7–28) (Table 2 and Figure 1), while the cumulative mean RGL of ChatGPT 4 was 17.9 (range, 13.7–32) (Table 3 and Figure 2). No significant difference was observed between the mean RGL of ChatGPT 3.5 and 4 (*p* = 0.95). Of the answers given by both ChatGPT 3.5 and ChatGPT 4, none (0%) were written at or below the recommended 8th grade reading level regardless of the readability test employed. The mean RGL of the answers given by ChatGPT 3.5 and ChatGPT 4 exceeded the 8th grade level by an average of 10.08 grade levels (*p* < 0.005) and 9.09 (*p* < 0.005), respectively. The mean FRES index ChatGPT 3.5 was 32.45 (range, 10–52), which is classified as “difficult”, while the mean FRES Index of ChatGPT 4 was 28.08 (range 9–47), which is classified as “very difficult”. A significant difference was observed between groups (*p* = 0.05).

## 4. Discussion

This study sought to evaluate the quality and readability of information provided by ChatGPT in relation to Anterior Cruciate Ligament (ACL) injuries and reconstruction. Our findings offer a significant insight into the role of A.I. tools like ChatGPT in patient education. While ChatGPT consistently delivers high-quality and balanced information regarding ACL injury and treatment, notable areas for concerns were identified regarding the lack of transparency in source citation and the accessibility of the provided content for the general public, owing to the complexity of language used.

OUT study used the DISCERN criteria to evaluate the quality of information provided by ChatGPT. Although statistical significance was not reached in the mean DISCERN Score between groups, it is noteworthy that ChatGPT 4 exhibited a significantly higher mean score per DISCERN criteria category. This suggests that the lack of significance in mean scores may be due our small sample size. Both programs scored highly in DISCERN criteria with ChatGPT 4 on the verge of an excellent. This indicates that AI tools are capable of providing information that is contextually relevant and highlights a potential role for ChatGPT in patient education in the future.

However, the consistent lack of source citation by both ChatGPT 3.5 and 4 should be a cause for concern among healthcare providers, one which makes the widespread adoption of ChatGPT as a tool for patient education at present unlikely. Furthermore, the ability of LLM to produced misleading or inaccurate answers, known as “Hallucinations” is another cause for concern. Hallucinations occur when a large language model generates false or misleading information and presents it as if it is factually correct. This occurs as LLMs are programmed to generate language that is grammatically and semantically correct within the context of a given prompt or question, but they do not always focus on the accuracy of the information provided. AI hallucinations can lead to misleading or false information generation, which can sometimes appear plausible. This is a particular concern in the realm of health literacy owing to its potential to mislead patients, perpetuate biases, and ultimately erode user trust by portraying unreliable or untrue information as fact. Before natural language processing tools, such as ChatGPT, can be recommended to patients by healthcare providers it is vital that they show improved transparency by referencing the sources of their information. Responses which are well-referenced serve to increase the credibility and trustworthiness of the information provided and allow physicians to recommend resources with improved confidence. While LLMs can generate highly individualized responses quickly, it should not come at the expense of the accuracy or reliability of the information provided.

Interestingly, source citation is available on other Large Language Models such as Perplexity A.I.; however, in this study, we chose to focus on the most popular Large Language Model in use among the general population, ChatGPT, to make our findings as applicable as possible. It is highly likely that future iterations of ChatGPT will have the ability to provide reliable sources for the information it provides, making it potentially a very useful tool in patient education.

One of the crucial findings of the study was related to the readability of the information provided by ChatGPT. The study found that the mean reading grade level (RGL) of the answers given by ChatGPT 3.5 was 18.08, and ChatGPT 4 had a mean RGL of 17.9. This was significantly higher than the recommended 8th-grade reading level for patient education materials (PEMs). In fact, the responses exceeded the 8th-grade level by an average of 10.08 and 9.09 grade levels for ChatGPT 3.5 and ChatGPT 4, respectively. Internet-based patient education materials in the field of Sports Medicine and Sports Orthopaedics have also been found to be written at a level which is far too complex for use in the general population, exceeding the recommended RGL by approximately four grade levels on average [24]. Our study found that ChatGPT generated responses which were at least nine grade levels above the recommended RGL. The findings in this regard are significant. They highlight a substantial gap between the reading capabilities of the general public and the complexity of the information provided by AI models like ChatGPT. To improve the accessibility of the responses provided by future iterations of ChatGPT, it is essential that answers are generated at significantly lower reading grade levels, ideally around the 6th grade level, to optimize readability for the general public.

The Flesch Reading Ease Index (FRES) of answers given by both ChatGPT 3.5 and 4 further supports this finding. The study classified the readability as “difficult” for ChatGPT 3.5 and “very difficult” for ChatGPT 4. This further underscores the challenge patients may face in understanding and utilizing the information presented to them, making the applicability and usability of ChatGPT in the general population at present questionable.

Our study’s findings have several important implications for healthcare. The results show the potential that AI may have a role in improving health literacy among our patients, allowing them to make informed treatment decisions. Healthcare providers and AI developers should, in future, work together to find a way to successfully convey complex of medical information at an appropriate level for patients’ reading abilities. The study highlights the key weaknesses in currently available AI models, demonstrating the importance of source citation in AI-generated responses. Properly referencing sources enhances the credibility of the information and provides patients and clinicians with the opportunity to independently verify the information provided to them. Additionally, the findings show that AI-models can be relied upon to emphasize the importance of shared decision-making between patients and healthcare providers. Patients want to be informed and involved in their healthcare decisions, and AI tools can play a valuable role in this process. The study acknowledges the potential of AI tools like ChatGPT in providing quick, an individualized information to patients. However, it suggests that further efforts are needed to optimize the readability of AI-generated content.

## 5. Conclusions

In conclusion, this study sheds light on the quality and readability of information provided by AI tools like ChatGPT in the context of ACL injuries and reconstruction. It underscores the importance of making healthcare information more accessible, accurate, and comprehensible for patients. The findings call for a collaborative effort among healthcare professionals and AI developers to ensure that AI tools align with the needs and capabilities of the patients they serve. As AI continues to play an increasing role in healthcare, addressing these issues is essential to empower patients in making well-informed decisions about their health.

We found that while the quality of the information provided by both ChatGPT versions is high, a glaring pitfall exists relating to the readability of the information provided. Despite the questions being framed at the average American reading level, both ChatGPT versions responded with answers which were highly complex. This suggests that while the information may be of high quality, its utility might be limited owing to the complexity of the answers it provides. Therefore, the potential role of AI models like ChatGPT in improving health literacy is currently hindered by the complexity of the answers it generates, which far exceed the level of comprehension of the general public.

While this study highlights the potential of AI tools like ChatGPT in patient education, it also demonstrates crucial areas for improvement, predominantly in terms of readability. It is paramount that developers address this limitation to fully harness the capabilities of AI in patient education, ensuring that information is not only accurate but also accessible to the masses.

## Figures and Tables

**Table 1:** DISCERN scores.

DISCERN Criteria	Mean Total DISCERN Score (95% C.I.)	Mean DISCERN Score per Criteria	Interrater Correlation Coefficient
ChatGPT 3.5	55.4 (range, 52.2–58.5)	3.46 (95% C.I. 3.40–3.54)	0.85
ChatGPT 4	62.09 (range 59.00–66.85)	3.86 (95% C.I. 3.79–3.93) *	0.91

* *p* ≤ 0.01. CI: 95%Confidence Interval.

**Table 2:** Reading grade level, ChatGPT 3.5.

Test	Valid N	Minimum	Maximum	Range	Mode(s)	Means
Flesch–Kincaid	33	10.2	18.4	8.2	14	14.7
Flesch Reading Ease	33	9	47	38	24; 26; 29; 30	28
Fry	33	13	17	4	17	16
Gunning Fog	33	11.7	19	7.3	19	17.2
Raygor Estimate	33	11	17	6	17	16
SMOG	33	12.5	19	6.5	16	16.6
Mean		11.23	22.90	11.67	17	18.08

SMOG: Simple Measure of Gobbledygook.

**Table 3:** Reading grade level, ChatGPT 4.

Test	Valid N	Minimum	Maximum	Range	Mode(s)	Means
Flesch–Kincaid	33	10.7	17.7	7	15	13.7
Flesch Reading Ease	33	10	52	42	34	32
Fry	33	11	17	6	17	16
Gunning Fog	33	10.6	19	8.4	12	14.2
Raygor Estimate	33	11	17	6	17	16
SMOG	33	12.8	18.7	5.9	13; 14; 15; 17	15.5
Mean		11.02	23.57	12.55	19.00	17.90

## Data Availability

The data supporting our findings can be requested from the investigating author.

## Appendix A. Questions

**No.**	**Frequently asked questions regarding ACL Injury and Treatment**
1.	Do I have to have surgery for my ACL?
2.	Should I choose to have ACL surgery?
3.	Who are the people who should consider ACL surgery?
4.	Can I avoid surgery if my ACL is torn?
5.	What are the different ways to do ACL surgery?
6.	Which kind of ACL surgery is the best: using tissue from the hamstrings, the kneecap area, the thigh muscle, or tissue from a donor?
7.	How do doctors usually do ACL surgery?
8.	What will happen after my ACL surgery?
9.	Can I play sports again after ACL surgery?
10.	How many days do I have to wait to play sports again after ACL surgery?
11.	When can I go back to work after my surgery?
12.	When can I start driving again after the surgery?
13.	Will I have to wear a cast after the ACL surgery?
14.	Will I need to wear a brace after the ACL surgery?
15.	Will I go to physical therapy after the ACL surgery?
16.	How long until I can jog in a straight line after the surgery?
17.	When can I play all kinds of sports again after the surgery?
18.	How long do I have to wait to drive a car again after the surgery?
19.	How successful is ACL surgery in letting people go back to sports or their usual activities?
20.	Will my new ACL be stronger than the one I had before the surgery?
21.	Is there a chance my ACL could tear again after the surgery?
22.	What problems might happen after ACL surgery?
23.	What problems might happen if I use tissue from my hamstrings for the surgery?
24.	What problems might happen if I use donor tissue for the surgery?
25.	What problems might happen if I use tissue from the kneecap area for the surgery?
26.	What problems might happen if I use tissue from the thigh muscle for the surgery?
27.	Will the ACL surgery be painful? Will I need medicine for the pain?
28.	Can I get arthritis after ACL surgery?
29.	What are the common problems after ACL surgery?
30.	How likely is it to tear my ACL again after it has been fixed in surgery?
31.	Can I get an infection from ACL reconstruction surgery?
32.	Does tearing my ACL make it more likely to get arthritis in the knee?
33.	Does having ACL surgery make it less likely to get arthritis in the knee compared to not having the surgery?

## Appendix B. Summary of Readability Formulae

**Readability Test**	**Score Type**	**Description**	**Formula**
Flesch–Kincaid Reading Grade Level	Grade Level	Designed for technical documents as part of the Kincaid Navy Personnel collection of tests. Applicable to a broad array of disciplines.	G = (11.8 × (B/W)) + (0.39 × (W/S)) − 15.59
Flesch–Kincaid Reading Ease	Index Score (0–100)	The standard test used by many US government agencies. Originally designed for newspaper readability. Best suited to school textbooks and technical documents. Scored from 0–100, with higher scores indicating easier readability.	I = (206.835 − (84.6 × (B/W)) − (1.015 × (W/S)))
The Raygor Estimate	Grade Level	Designed for most text, including literature and technical documents	Calculated using the mean number of sentences and long words (≥6 characters) per 100 words, which are plotted on to a RE Graph, where their intersection determines RGL.
Fry	Grade Level	Designed for most texts, including technical documents and literature, across a range of levels, from primary school to university level. Best suited to educational texts.	Calculated using the mean number of sentences and syllables per 100 words, which are plotted on to a Fry Graph, where their intersection determines RGL.
SMOG	Grade Level	Assesses for 100% comprehension, whereas most formulae test for roughly 50–75% comprehension. Applicable to secondary age (4th grade to college) level texts. Most accurate when applied to documents ≥30 sentences in length.	G = 1.0430 × √C + 3.1291
FORCAST	Grade Level	The only test not designed for running narrative. Developed to assess US army technical manuals and forms.	G = 20 − (M/10)
Gunning Fog	Grade Level	Applicable to numerous disciplines. Originally designed for American businesses to improve the readability of their writing.	G = 0.4 × (W/S + ((C*/W) × 100))
		G = grade level; B = number of syllables; W = number of words; S = number of sentences; RGL = reading grade level; I = Flesch Index Score; RE = Raygor Estimate; SMOG = Simple Measure of Gobbledygook; C = complex words (≥3 syllables); E = predicted Cloze percentage = 141.8401 − (0.214590 × number of characters) + (1.079812 × S); M = number of monosyllabic words; C* = complex words with exceptions, including proper nouns, words made three syllables by the addition of “ed” or “es”, and compound words made of simpler words.	

## References

[1] Herzog M.M., Marshall S.W., Lund J.L., Pate V., Spang J.T. (2017). Cost of Outpatient Arthroscopic Anterior Cruciate Ligament Reconstruction among Commercially Insured Patients in the United States, 2005–2013. Orthop. J. Sports Med..

[2] Sanders T.L., Kremers H.M., Bryan A.J., Larson D.R., Dahm D.L., Levy B.A., Stuart M.J., Krych A.J. (2016). Incidence of Anterior Cruciate Ligament Tears and Reconstruction. Am. J. Sports Med..

[3] Kohn L., Rembeck E., Rauch A. (2020). Verletzung des vorderen Kreuzbandes beim Erwachsenen. Orthopade.

[4] Clatworthy M., Fulcher M., Chang K., Young S.W. (2019). Marked increase in the incidence of anterior cruciate ligament reconstructions in young females in New Zealand. ANZ J. Surg..

[5] Ardern C.L., Sonesson S., Forssblad M., Kvist J. (2017). Comparison of patient-reported outcomes among those who chose <scp>ACL</scp> reconstruction or non-surgical treatment. Scand. J. Med. Sci. Sports.

[6] Grevnerts H.T., Krevers B., Kvist J. (2022). Treatment decision-making process after an anterior cruciate ligament injury: Patients’, orthopaedic surgeons’ and physiotherapists’ perspectives. BMC Musculoskelet. Disord..

[7] Wang C., Li H., Li L., Xu D., Kane R.L., Meng Q. (2013). Health literacy and ethnic disparities in health-related quality of life among rural women: Results from a Chinese poor minority area. Health Qual. Life Outcomes.

[8] Kirsch I., Jungeblut A., Jenkins L., Kolstad A. (1993). Adult Literacy in America: A First Look at the Results of the National Adult Literacy Survey.

[9] Cotugna N., Vickery C.E., Carpenter-Haefele K.M. (2005). Evaluation of Literacy Level of Patient Education Pages in Health-Related Journals. J. Community Health.

[10] Weiss B.D., Blanchard J.S., McGee D.L., Hart G., Warren B., Burgoon M., Smith K.J. (1994). Illiteracy among Medicaid Recipients and its Relationship to Health Care Costs. J. Health Care Poor Underserved.

[11] Brega A.G., Freedman M.A.G., LeBlanc W.G., Barnard J., Mabachi N.M., Cifuentes M., Albright K., Weiss B.D., Brach C., West D.R. (2015). Using the Health Literacy Universal Precautions Toolkit to Improve the Quality of Patient Materials. J. Health Commun..

[12] Hautala G.S., Comadoll S.M., Raffetto M.L., Ducas G.W., Jacobs C.A., Aneja A., Matuszewski P.E. (2021). Most orthopaedic trauma patients are using the internet, but do you know where they’re going?. Injury.

[13] Doinn T.Ó., Broderick J.M., Abdelhalim M.M., Quinlan J.F. (2021). Readability of Patient Educational Materials in Pediatric Orthopaedics. J. Bone Jt. Surg..

[14] Halverson J.L., Martinez-Donate A.P., Palta M., Leal T., Lubner S., Walsh M.C., Strickland J.S., Smith P.D., Trentham-Dietz A. (2015). Health Literacy and Health-Related Quality of Life Among a Population-Based Sample of Cancer Patients. J. Health Commun..

[15] Al Sayah F., Qiu W., Johnson J.A. (2016). Health literacy and health-related quality of life in adults with type 2 diabetes: A longitudinal study. Qual. Life Res..

[16] Hurley E.T., Crook B.S., Lorentz S.G., Danilkowicz R.M., Lau B.C., Taylor D.C., Dickens J.F., Anakwenze O., Klifto C.S. (2023). Evaluation High-Quality of Information from ChatGPT (Artificial Intelligence—Large Language Model) Artificial Intelligence on Shoulder Stabilization Surgery. Arthrosc. J. Arthrosc. Relat. Surg..

[17] Currie G.M. (2023). GPT-4 in Nuclear Medicine Education: Does It Outperform GPT-3.5?. J. Nucl. Med. Technol..

[18] Khair M.M., Ghomrawi H., Wilson S., Marx R.G. (2018). Patient and Surgeon Expectations Prior to Anterior Cruciate Ligament Reconstruction. HSS J..

[19] Gilat R., Cole B.J. (2023). How Will Artificial Intelligence Affect Scientific Writing, Reviewing and Editing? The Future is Here …. Arthrosc. J. Arthrosc. Relat. Surg..

[20] Feucht M.J., Cotic M., Saier T., Minzlaff P., Plath J.E., Imhoff A.B., Hinterwimmer S. (2016). Patient expectations of primary and revision anterior cruciate ligament reconstruction. Knee Surg. Sports Traumatol. Arthrosc..

[21] OleanderSoftware (2021). Readability Studio 2021: Professional Edition (Version 2021).

[22] Weis B.D. (2003). Health Literacy: A Manual for Clinicians.

[23] Sudore R.L., Yaffe K., Satterfield S., Harris T.B., Mehta K.M., Simonsick E.M., Newman A.B., Rosano C., Rooks R., Rubin S.M. (2006). Limited literacy and mortality in the elderly: The health, aging, and body composition study. J. Gen. Intern. Med..

[24] Doinn T.Ó., Broderick J.M., Clarke R., Hogan N. (2022). Readability of Patient Educational Materials in Sports Medicine. Orthop. J. Sports Med..

